# Performance of GPT-4o and Claude in Medical Ethics Scenarios: Comparative Study

**DOI:** 10.2196/70199

**Published:** 2026-07-23

**Authors:** Karishma R Desai, Anna L Gorsky, Nicole A Zelenski

**Affiliations:** 1 Division of Hand Surgery, Department of Orthopaedic Surgery, Emory University, 80 Jesse Hill Jr Drive SE, Atlanta, GA, 30303, United States, 1 404-778-1550

**Keywords:** medical education, ethics, legal, ChatGPT, artificial intelligence, medical student, orthopedics, Claude, resident, health

## Abstract

**Background:**

The emergence of AI technology has sparked curiosity regarding the capabilities of large language models (LLMs) in the field of medicine. Minimal research exists regarding the proficiency of various AI models in ethics scenarios, specifically in specialty-based scenarios.

**Objective:**

This study aimed to compare the performance of GPT-4o and Claude Sonnet 4 on ethics questions with that of medical students and orthopedic residents.

**Methods:**

A total of 200 ethical or legal scenario questions were randomly selected from question banks targeted for third- and fourth-year medical students (UWorld, AMBOSS) and orthopedic residents (OrthoBullets). Questions at the medical student level were exclusively text-based, while resident-level questions included text-based questions accompanied by images. Each question was entered identically into each AI model 3 separate times. If answers varied between trials, the answer provided most frequently by the model was used as the selected answer.

**Results:**

GPT-4o correctly answered 140 (70%) of 200 questions, which was similar to the average human test taker score of 71% (~142/200 questions). Claude correctly answered 180 (89%) questions, a score greater than that of human test takers and significantly better than GPT-4o (*P<*.001). Claude scored significantly higher than GPT-4o in almost all question categories. GPT-4o provided different responses to identically worded trials for 27 (21%) of 130 general questions and 3 (4%) of 70 orthopedic questions (*P=*.002), while Claude did not have a significant difference in variability between these 2 groups (general: 16/130, 12% vs orthopedic: 3/70, 4%; *P*=.06). Neither GPT-4o or Claude showed a significant difference in the rate of most common incorrect response selection between question categories. Claude showed no significant difference in the rate of most common incorrect response selection between question categories.

**Conclusions:**

These results suggest that GPT-4o can potentially answer both general and specialty-specific ethical questions with similar proficiency to sample groups of both medical students and orthopedic residents, while Claude AI performs significantly better than both humans and GPT-4o. Variables such as AI model framework and training data may drive the observed difference in performance, but the exact cause cannot be definitively isolated without intentional testing. Therefore, further research is needed to ensure safety by minimizing output variability before integrating AI as a patient-facing resource.

## Introduction

Interpersonal skills are an invaluable necessity for health care providers. Impactful medical practice requires the ability to adapt treatment plans to a patient’s unique preferences, which is founded on the development of the patient-provider relationship. Numerous studies have shown that effective physician communication is positively correlated with patient satisfaction [1] and positive health outcomes [2]. Patient involvement in their own medical care, often termed “shared decision-making,” has also been observed to correlate with increased overall and disease-specific quality of life scores in orthopedic patients [3]. The now near-ubiquity of internet usage has resulted in patients frequently consulting online materials for additional medical information. Nearly all American households had internet access in 2021 [4], and recent Centers for Disease Control and Prevention data suggest that more than half of adults (58.5%) use the internet for medical advice [5]. This trend is also mirrored internationally, with 1 study showing that 64.3% of Lebanese patients consulted web resources for acute symptoms, with 19.2% seeking web information before physician consultation [6].

The increasing popularity of AI technologies has introduced a new potential source for internet-accessible medical information. Large language models (LLMs), such as GPT-4o (OpenAI), are currently being evaluated for their ability to play a role in health care. AI has been shown to accurately process medical knowledge and show proficiency at the level of third- and fourth-year medical students in preparation for the United States Medical Licensing Examination [7-9]. In addition, GPT-4o has been challenged with postgraduate examination materials, including preparation materials for the American Board of Surgery In-Training Examination [10,11], neurology specialist examinations [12], and the Orthopaedic In-Training Examination [13,14] and, in most cases, has performed at the level of a human medical trainee. Recent exploration has shown that ChatGPT models can also directly respond to patient questions about common conditions across a variety of specialties with generally satisfactory answers [15-17].

Nevertheless, integration of AI into health care settings remains controversial due to a lack of clarity surrounding its strengths and limitations, and it is unclear whether AI can be safely recommended to serve as a substitute or even as an adjunct to physician consultation [18]. The “soft skills” of medical training include compassion and empathetic communication, which are essential to identifying and navigating the nuances present within each individual patient interaction. Although AI technologies have shown some capability in correctly reproducing technical knowledge and answering patient questions, their capability to identify and process user emotion is not well understood.

LLMs are modeled on neural networks, in which units or “tokens” of data are connected and linked by associations, which are strengthened or weakened based on the presence of patterns within training data [19]. Essentially, the models use the patterns identified from their training data to generate outputs that are the most probabilistically likely “correct” responses [20]. Transformer architecture is the modern evolution of neural networks and uses “self-attention” to consider and weight tokens in relation to each other [21]. Through the training process, the model adapts the level of attention given to various tokens to identify the most relevant components of the input and better generate more relevant responses in the given context [21]. Ultimately, transformer architecture improves the statistical predictive accuracy of LLMs, especially in the processing of large volumes of inputs, by more accurately weighing the relevance of input tokens [21].

Training can occur through the establishment of parameters, which guide the model in processing data inputs to promote or oppose specific outputs; positive reinforcement of a specific association teaches the model that the reinforced logic has an increased likelihood of leading to the correct output [22]. Feedback can be provided to the model based on the final output (outcome supervision) or throughout the logical process (process supervision), and the model applies this feedback and recalculated probabilities to direct the processing of new data [23]. Therefore, the veracity of AI-generated outputs is dependent directly on the quality and accuracy of the training data and subtleties within the inputs themselves, rather than solely on known truths [24]. As a result, AI models have been documented to “hallucinate” or fabricate information and present it convincingly as fact [25]. Researchers in one study found that a number of inconsistencies and hallucinations were noted when questions on sensitive or obscure topics were presented to several AI models [26], supporting the hypothesis that AI performance declines when available data are limited. “Factuality” hallucinations, when models report information that violates known facts, and “faithfulness” hallucinations, when models deviate from the information provided in the given input, are theorized to occur due to inconsistent training and model evaluation metrics [27].

Despite numerous studies evaluating AI’s accuracy in answering questions based on empiric knowledge, the literature supporting AI’s performance in health care–based medical ethical, legal, and social scenarios is limited and inconsistent. Some reports demonstrate that LLMs can answer ethical scenario questions [28] and respond to patient clinical questions with answers that are rated positively for empathy by human raters [29,30], while others found ChatGPT models performed worse on ethics questions compared to medical trainees [10,31,32]. When subjected to emotion-related testing, AI demonstrated the ability to correctly identify and manage emotions but struggled when associating emotions to rationale and scored lower than the human average on the “using emotions to facilitate thought” section of the examination [33].

Variant AI models have been designed to address the general safety and ethical concerns surrounding the outputs produced by LLMs. One such model is Claude, created by Anthropic, which describes it as “helpful, honest, and harmless” [34]. Claude was developed using a novel “Constitutional AI” method, meaning that instead of using human feedback during the training process, feedback is instead provided by the AI model itself, guided by a provided set of “constitutional principles” [35]. During the supervised training process, the model is asked prompts that will purposefully generate a “harmful” output, for example, asking for instructions on how to break into a house. The model is then asked to critique the output based on a constitutional principle (“how is the last response unethical or illegal”) and then to revise the output according to the critique (“rewrite the response to remove unethical or illegal content”) [36]. In the reinforcement learning or unsupervised phase, the model is asked to generate multiple responses to a prompt, and a different feedback model is asked to choose one of the responses also based on a constitutional principle (“which of these responses is the most ethical and legal?”) [36]. Therefore, humans are not providing feedback for each model response themselves, but instead instructing a “teaching” AI to guide the “learning” model to align with specific rules, or constitutional principles. Anthropic sources its constitutional principles from a number of ethic-centered documents, including the UN Declaration of Human Rights, as well as principles developed by other AI laboratories and Anthropic researchers themselves during model training to discourage harm, aggression, offense, violence, and so on [35]. Although Claude is intentionally designed with ethics and safety in mind, there are no available studies specifically exploring Claude’s performances in ethics-related health care scenarios.

Throughout medical school and postgraduate medical training, learners are taught how to manage complex social issues and interpersonal interactions with a high degree of emotional intelligence and empathy. Even ethical dilemmas, which are addressed using well-defined ethical principles, must be navigated within the larger context of patient emotion. Medical training examinations often use questions about end-of-life discussions, patient safety, communication strategies, and informed consent to assess these intangible skills. This study aimed to evaluate the ability of GPT-4o and Claude Sonnet 4 to answer health care–based ethical medical board–style questions accurately and consistently. We hypothesized that GPT-4o and Claude will differ in performance between question levels and categories.

## Methods

All questions used for testing were scenario-based multiple-choice questions for which there was only one correct answer. Medical student–level questions were sourced from the UWorld Step 2 CK question bank and AMBOSS, 2 resources designed to prepare third- and fourth-year medical students for the Step 2 benchmark examination. The UWorld system allows for question sourcing from selected subject categories. One category of interest was the broad “Social Sciences (Ethics/Legal/Professional)” category, which involves the subcategories of Communication and Interpersonal Skills, Healthcare Policy and Economics, Medical Ethics and Jurisprudence, Patient Safety, and System-based practice and Quality Improvement. The subcategory “Communication and Interpersonal Skills” was also specifically selected as a second source of questions to assess whether performance differed on questions specifically relating to interpersonal communication scenarios, which often require interpretation of patient tone and social cues. Both types of questions typically begin with patient vignettes. Ethics or legal questions often ask, “Which of the following is the best next step in management of this patient?” in scenarios that require the physician to make a decision about common clinical dilemmas involving end-of-life care, next-of-kin decision-making power, or consent for emergent procedures. Interpersonal questions typically present a patient scenario regarding a sensitive subject such as alcohol dependence before asking, “which of the following is the most appropriate response to this patient?” The test taker is given several different responses as answer choices that vary in tact and counseling advice. Individual question specifics cannot be reproduced, as these question banks are copyright protected; however, these questions are designed to imitate the style of questions typically asked on the USMLE Step 2 CK examination. An example of this question’s style provided by the USMLE is provided as follows [37]:

A 67-year-old man is evaluated in the intensive care unit. He has end-stage pancreatic cancer and was hospitalized 3 days ago for treatment of pneumonia. Respirations are 6/min. Pulse oximetry on 100% oxygen by face mask shows an oxygen saturation of 78%. Examination shows feeble respiratory efforts; he is using accessory muscles of respiration. On mental status examination, the patient is oriented to person but not to place or time. If the patient is not endotracheally intubated and mechanically ventilated, he will die within hours. His wife says the patient recently told her that he would never want mechanical ventilation, but they never completed paperwork regarding his wishes. His daughter insists that he be mechanically ventilated. Which of the following is the most appropriate action for the physician to take?

- Perform endotracheal intubation and begin mechanical ventilation
- Perform endotracheal intubation and then consult the hospital ethics committee regarding mechanical ventilation
- Perform endotracheal intubation only
- Provide palliative therapy only
- Seek a court order to assign a legal guardian

As these ethical dilemmas are typically framed in the context of emotional situations, the complexity and nuance of addressing these issues often stem from the application of concrete ethical principles to emotionally charged and sensitive scenarios. Therefore, identification of the correct or “most appropriate response” requires not only an understanding of ethical principles but also the ability to apply these principles in a manner that is sensitive to the patient. The interpersonal communication questions were included to assess not only LLM performance in ethical scenarios but also to assess whether there was unique accuracy or inaccuracy in the skill of identifying the textbook “most appropriate answer.”

The UWorld software allows users to create custom tests based on subject categories with up to 40 questions per test. The individual questions available in each subject category are not visible to test takers due to the nature of the test bank, and therefore, the questions included in each test could not be individually predetermined. A 40-question test was generated from each category described previously—one test using questions from the broader “Social Sciences (Ethics/Legal/Professional) category” and one test using questions exclusively from the “Communication and Interpersonal Skills” subcategory.

As the UWorld questions were not individually selected prior to test generation, 15 questions were duplicated between the 40-question sets. The duplicated questions were not excluded as the average test taker performance score is generated based on the full 40-question set, and therefore, inclusion of all questions was necessary to compare LLM performance to human performance. This method ensured that each test would be representative of tests encountered by human testers.

The AMBOSS question bank similarly allows the creation of tests involving randomly generated questions within a subcategory, although with a higher maximum of 50 questions. The AMBOSS “Legal Medicine and Ethics” category was the only equivalent available subcategory to test the ethics-related medical competencies, and therefore, a test of 50 randomly selected questions from the AMBOSS Legal Medicine and Ethics discipline was generated. The format of these questions mirrored those of UWorld questions in that they are designed to imitate the style of questions asked on the USMLE Step 2 examination.

Another area of interest was whether model performance would vary in specialty-specific contexts. By testing questions written at a level for medical residents, LLM performance can be evaluated in scenarios at higher levels of medical complexity. This aim of the study was treated as completely exploratory, and causal relationships should not be inferred. Orthopedics was chosen as the specialty due to the author’s access to Orthobullets, an online resource intended to prepare orthopedic residents for the Orthopaedic In-Training Examination. This question bank similarly allows the generation of subject-specific tests and provides 2 question categories relevant to this study “Ethics in Orthopaedics Practice” and “Legal Considerations in Orthopaedic Practice.” A total of 70 questions were available: 21 from the Ethics category and 49 from the Legal category. All questions were included in this study. The ethics questions required test takers to identify the most ethical course of actions in orthopedic patient–specific scenarios involving potential financial conflicts of interest with research studies and transfers of value from pharmaceutical or medical device representatives, obtaining interpretation services for a non–English-speaking patient, and appropriate marketing practices. Legal consideration questions involved scenarios in which test takers were required to identify the most appropriate way to obtain consent in emergent situations or manage medical or surgical errors, altering the medical record of surgical errors, or preoperative protocol management, and professionalism violations.

All questions from UWorld and AMBOSS were text-based by nature of the test banks, while Orthobullets questions included text-only questions as well as questions with supporting images. The inclusion of questions involving images introduced a potential for confounding, but it was thought that analysis performed at the question bank–level would illuminate whether LLM performance varied by question bank. Only 7 questions of the 70 sourced from Orthobullets included images, and this volume was not considered sufficient to draw conclusions about varying performance based on question modality. For all question banks, the software provided a metric of the “average” score earned by test takers on the same test, and this is the benchmark used in this study as the “human test taker” performance metric. It is important to note that the multiple-choice questions used are not validated measures of ethical reasoning or empathetic reasoning but simply an available tool to compare performance to that of human test takers. Information on the specifics of how these benchmarks are calculated is not publicly available for any of the question banks. Therefore, questions could not be excluded in our study from the tests generated by the software, as the average human test taker benchmark could not be manually calculated.

**Figure 1.**
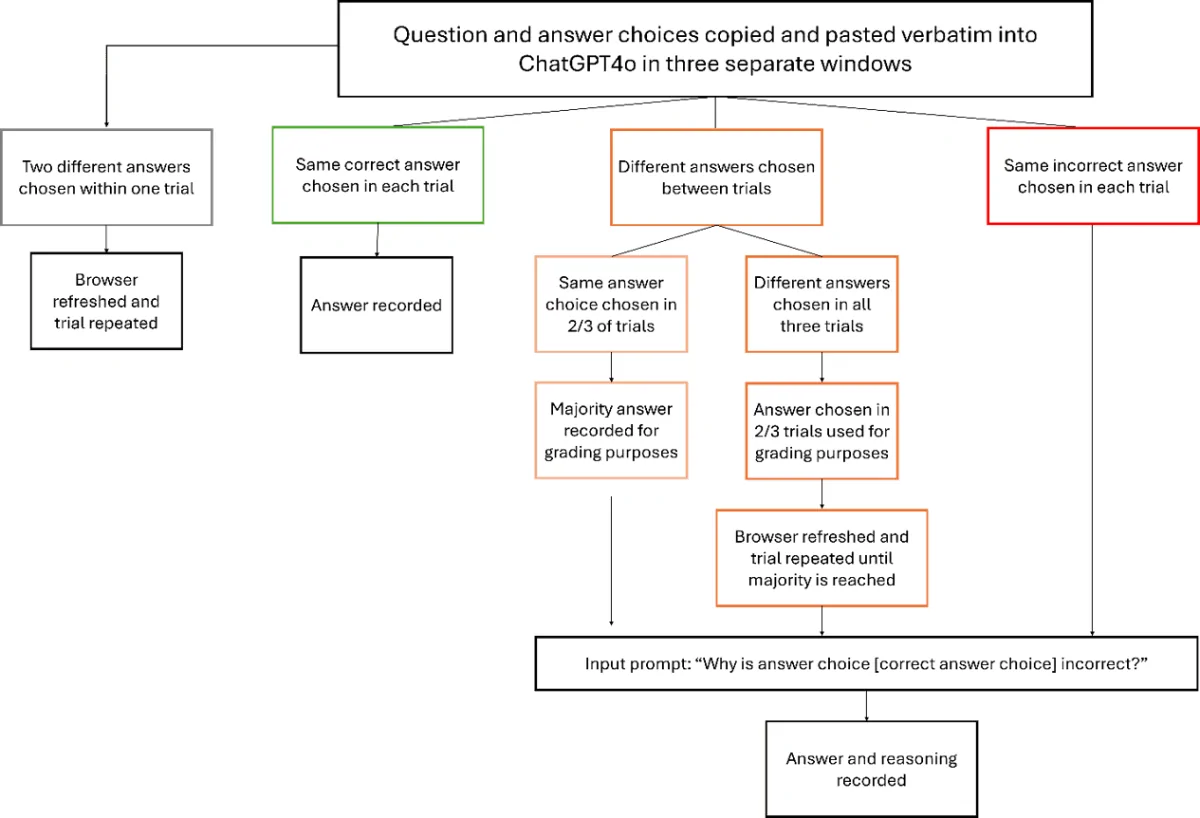
Testing method, repeated for each question.

A zero-shot approach was used to conduct the trials; no training or prompt engineering was performed to provide context to the models [38]. Instead, the free, publicly available version of GPT-4o was opened in a browser window, a singular question and its corresponding answer choices were copied and pasted verbatim as the input, and the resulting selection was recorded. If any images were provided in accompaniment to the question stem, these were also provided to the chatbot in the same input as the question stem. To prevent bias or learning for subsequent trials, a new, unique browser window was opened, and the above, zero-shot approach was repeated for each trial. If the provided answer varied between trials, the answer generated in the majority of trials was recorded as GPT-4o’s selected response. Three trials were performed for each question, and additional trials were conducted as needed to achieve a majority response if there was no consensus between the original 3 trials. Typically, GPT-4o provided reasoning for its selection without additional prompting. If the incorrect answer was chosen, the reasoning behind the incorrect choice was recorded. If no reasoning was provided, GPT-4o was then prompted with “Why is answer choice [the correct answer] incorrect?” If answers varied between trials, GPT-4o’s evaluation of the correct answer choice, incorrect answer choice, and previously selected answer choices were recorded. All questions tested required the selection of a singular answer choice. On the occasions when GPT-4o selected 2 answer choices as the correct answer, the browser was refreshed, and the trial was repeated until GPT-4o provided only one answer choice. The testing procedure can be visualized in Figure 1.

For all questions, the question banks also provided individual test taker benchmarks on the percentage of subscribers that selected each individual answer choice. It is important to note that the exact method of calculation for this metric was also not publicly available for each question bank. If GPT-4o selected the incorrect answer, it was noted whether or not its selection was the incorrect answer choice most popularly chosen by humans.

The same zero-shot testing method was used for Claude Sonnet 4. Claude users are required to pick a category of interest on sign-in. The “learning and studying” option was selected for all trials included in this study, but no additional prompt engineering or configurations were made to this chatbot.

Statistical analysis to compare the AI model’s overall performance against human performance was performed using SPSS (version 31.0.0.0 (117); IBM) with one-sample proportion tests, and McNemar tests were used to compare performance between models. Chi-square and Fisher exact tests were used to analyze model ability to select the most popular incorrect response as well as the frequency of varying responses to identical questions.

## Results

It is unclear how the question banks derived the reported human performance averages and whether these scores are truly representative of the true population of medical test takers. Therefore, statistical inferences are not valid, and comparisons between LLM and human performance are reported purely descriptively. The performance of GPT-4o was similar to the average score of the question banks’ human test takers (71% vs 70%). GPT-4o correctly answered 89 (68%) of 130 general medical student–level questions, and 51 (73%) of 70 orthopedic resident–level questions with no significant difference in performance, compared to the human averages of 71% and 73%, respectively. Claude answered 180 (90%) of 200 total questions correctly, performing better than the human average (89% vs 71%) and significantly better than GPT-4o (89% vs 70%; *P*<.001). Claude’s score was higher than the provided human average on all question banks. Claude also performed significantly better than GPT-4o on all question categories except the UWorld Social Sciences and Orthobullets Ethics questions; results and statistical conclusions are summarized in Table 1, with the number of questions correct and correlated weighted score as provided by each test bank.

**Table 1.** Correctly answered by human test takers, GPT-4o, and Claude per question set^a^.

Question set	Human test taker score, n/N (%)	GPT-4o score, n/N (%)	Claude score, n/N (%)	Between-model comparison, *P* value
UWorld All Social Sciences	~27/40 (68)	27/40 (68)	33/40 (83)	.14
UWorld Interpersonal-Specific	~28/40 (69)	30/40 (75)	36/40 (90)	.03^b^
AMBOSS	~36/50 (72)	32/50 (64)	46/50 (92)	<.001^b^
Medical student–level or nonspecific questions	~92/130 (71)	89/130 (68)	115/130 (88)	<.001^b^
Orthobullets Ethics	~15/21 (73)	14/21 (67)	17/21 (81)	.25
Orthobullets Legal	~36/49 (73)	37/49 (76)	48/49 (98)	<.001^b^
Resident-level/orthopedic questions	~51/70 (73)	51/70 (73)	65/70 (92)	<.001^b^
Overall performance	~142/200 (71)	140/200 (70)	180/200 (89)	<.001

a Aggregate question categories (medical student, orthopedic resident, and overall) performance were calculated using averages of human test taker percentage scores. “Between model comparison” represents the statistical significance of exclusive comparison between GPT-4o and Claude performance.

b Statistically significant results (P<.05).

Question banks only provided average human test taker score in percentages, and equivalent numbers of correctly answered questions were calculated based on the provided percentage correct and total number of questions used.

GPT-4o answered 60 (30%) of 200 total questions incorrectly. Of these 60 questions, the most popular incorrect response was chosen 37 (62%) times. Analysis did not show a significant difference in the frequency that GPT-4o chose the most popular incorrect answer between both UWorld general social sciences and interpersonal-specific sets (*P*=.07), UWorld (general and interpersonal) and AMBOSS (legal medicine and ethics) sets (*P*=.33), Orthobullets Ethics and Legal sets (*P*=.22), or medical student general and orthopedic resident–level (UWorld/AMBOSS and Orthobullets) sets (*P*=.46; Table 2).

**Table 2.** Incorrectly answered questions per question set for which GPT-4o and Claude selected the incorrect answer most commonly chosen by human test takers.

Question set	GPT-4o^a^, n/N (%)	Claude^b^, n/N (%)
UWorld All Social Sciences	6/13 (46)	5/7 (71)
UWorld Interpersonal-Specific	9/10 (90)	4/4 (100)
AMBOSS	9/18 (50)	3/4 (75)
Orthobullets Ethics	6/7 (86)	4/4 (100)
Orthobullets Legal	7/12 (58)	1/1 (100)

a Total: 37/60 (62%).

b Total: 17/20 (85%).

Claude incorrectly answered 20 (10%) of 200 total questions. Analysis using Fisher exact test did not show a significant difference in the frequency that Claude chose the most popular incorrect answer between both UWorld general social sciences and interpersonal-specific sets (*P*=.49), UWorld (general and interpersonal) and AMBOSS (legal medicine and ethics) sets (*P*>.99), Orthobullets Ethics and Legal sets (*P*>.99) or medical student general and orthopedic resident–level (UWorld/AMBOSS and Orthobullets) sets (*P*>.99; Table 2).

GPT-4o varied answers on 30 (15%) of 200 total questions, while Claude varied answers on 19 (10%) of the 200 total questions. There was no significant difference between the frequency of varying answers between GPT-4o responses for both the UWorld general social sciences and interpersonal-specific sets (*P*=.23) UWorld (general and interpersonal) and AMBOSS (legal medicine and ethics) sets (*P*=.25), and Orthobullets Ethics and Legal sets (*P*=.90). However, GPT-4o produced variable answers between trials significantly less frequently for orthopedic resident–level questions than for medical student general questions (*P*=.002; Table 3).

**Table 3.** Questions per question set for which AI models changed answers between trials.

Question set	GPT-4o^a^, n/N (%)	Claude^b^, n/N (%)
UWorld All Social Sciences	5/40 (13)	5/40 (13)
UWorld Interpersonal-Specific	9/40 (23)	6/40 (15)
AMBOSS	13/50 (26)	5/50 (10)
Orthobullets Ethics	1/21 (5)	1/21 (5)
Orthobullets Legal	2/49 (4)	2/49 (4)

a Total: 30/200 (15%).

b Total: 19/200 (10%).

Claude did not significantly differ responses for both UWorld general social sciences and interpersonal-specific sets (*P*=.75), UWorld (general and interpersonal) and AMBOSS (legal medicine and ethics) sets (*P*=.53), and Orthobullets Ethics and Legal sets (*P*=.90), or between medical student general questions and orthopedic resident–level questions (*P*=.06).

There was no significant difference in variability of responses between the 2 models overall (*P*=.09), for medical student general questions (*P*=.07) or for orthopedic resident–level questions (*P*>.99).

When both models varied their answers, the explanations between various sessions were often contradictory; one session of the model would provide reasoning as to why an answer choice was correct, and a different session of the model would cite the same reasoning as the reason that answer choice was incorrect. Examples of contradictory reasoning are provided in Tables 4 and 5.

**Table 4.** Examples of contradictory reasoning from GPT-4o.

Question set	Question scenario	Trial 1 reasoning	Trial 2 reasoning
UWorld All Social Sciences	Next best step in management of a pediatric patient refusing chemotherapy against the wishes of her family	“The best next step: Before any decisions are made regarding treatment, it is crucial to assess whether the child has the cognitive and emotional maturity to make an informed decision. In cases of disagreement between family members, the healthcare team, and the patient, an ethics committee meeting may be helpful. However, this would be premature until the child’s capacity is assessed. It is better to first evaluate whether the child can make her own decision before escalating the situation to an ethics committee.”	“An ethics committee can help mediate the disagreement between the patient, the parents, and the medical team. It allows for a structured discussion of the ethical principles involved, including the child’s autonomy, the parental authority, the treatment’s potential benefits and burdens, and the patient’s best interests. An ethics consultation provides a thorough review and a neutral perspective on how to balance these competing factors. [Requesting evaluation of patient for decision-making capacity] seems reasonable, but it’s crucial to note that at 8 years old, a child is unlikely to have the legal capacity to make independent medical decisions.”
UWorld Interpersonal-Specific	Next best step in management of a patient presenting after office hours for a nonemergent concern	“Healthcare providers must set reasonable boundaries around after-hours care. While the patient may be dissatisfied with the timing, the clinic’s normal operational hours should be respected unless there is an urgent need (such as signs of severe infection, anaphylaxis, etc), which does not seem to be the case here. ...The patient has a non-urgent concern (itchy rash) that was already evaluated a week ago, and it doesn’t appear to be an emergency... A rash that was already examined a week ago and treated for dry skin is unlikely to require immediate intervention.”	“While it is important to adhere to office hours, patient safety and care should always take precedence. The physician should evaluate the patient to assess the severity of her symptoms and provide appropriate care, especially since rashes and skin conditions can sometimes escalate quickly.”
AMBOSS	Most appropriate action following parent refusal of chemotherapy and decision to pursue supportive care for pediatric patient	“The parents’ decision should be respected after they have been fully informed about the potential consequences. If they choose to forgo chemotherapy, supportive care should be provided, ensuring the child’s comfort.”	“While it is important to respect parental wishes, the physician has an ethical responsibility to ensure that the child receives appropriate treatment. In this case, supportive care alone is not appropriate for intermediate-risk neuroblastoma, as it could lead to significant harm or death.”
Orthobullets Ethics	Analysis of orthopedic patient complaints	“Lack of empathy can certainly affect a patient’s overall experience, but it’s generally not the top complaint compared to logistical frustrations, such as long waits for visits or difficulties with prescription management.”	“In summary, long waits for visits are an issue but not the most frequent complaint that patients tend to focus on when evaluating their orthopaedic care. The lack of empathy is more often identified as a key source of dissatisfaction in this specialty.”
Orthobullets Legal	Analysis of patient-physician interaction	“Research suggests that, on average, a physician will allow the patient to speak for about 60 seconds (1 minute) after asking an open-ended question, before they typically redirect or intervene.”	“On average, patients speak for about 23 seconds before the physician redirects the conversation or takes control of the dialogue, often in order to gather more focused or specific information.”

**Table 5.** Examples of contradictory reasoning from Claude.

Question set	Question scenario	Trial 1 reasoning	Trial 2 reasoning
UWorld All Social Sciences	Next best step in management of an unconscious hypotensive patient receiving blood transfusion. His next-of-kin requests the transfusion be stopped due to religious purposes; no advance directive available	“The answer is: [Continuing transfusion until patient is stable] The patient is in immediate life-threatening condition Treatment was initiated under emergency doctrine Without written documentation, the physician cannot be certain of the patient’s specific wishes The [next-of-kin’s] statement, while important, is not sufficient legal documentation in this emergency Once stabilized, there would be time to obtain proper documentation or ethics consultation This approach prioritizes the irreversible nature of death while still respecting the process of determining patient wishes This represents the standard approach in emergency medicine: when in doubt and life is immediately threatened, preserve life while working to clarify the patient’s true wishes through proper channels.”	“Option [Continuing transfusion until patient is stable] fundamentally violates the principle of patient autonomy and informed consent for several reasons: Autonomy doesn’t pause for medical convenience: A patient’s right to refuse treatment doesn’t become suspended just because they’re unstable. If we accepted this logic, we could override any patient’s wishes whenever their condition was serious enough. Temporal problem: “Until stabilized” creates an arbitrary endpoint that prioritizes medical goals over the patient’s values. Stabilization could take hours, days, or longer - during which time the patient’s clearly expressed wishes (through his wife) are being violated. ... Slippery slope: If physicians could override known patient wishes whenever they deemed the patient “too unstable,” it would essentially nullify the right to refuse treatment in emergency situations. The key ethical point: Patient autonomy and the right to refuse treatment based on religious beliefs doesn’t diminish based on the severity of the medical situation. Once the patient’s wishes are reliably known through an appropriate surrogate, those wishes should be respected immediately, not delayed for medical convenience.”
UWorld Interpersonal- Specific	Asking what issue is most likely for a patient demonstrating poor health literacy	“This patient demonstrates concerning gaps in health literacy - she has a major misunderstanding about her condition and doesn’t know her current medications. Poor health literacy is strongly associated with decreased adherence to follow-up care, missed appointments, and poor engagement with the healthcare system. Despite being educated [teacher], health literacy and educational attainment don’t always correlate. Her poor medication knowledge and health literacy might actually lead to underutilization rather than overuse of services. The patient’s demonstrated poor health literacy [major misunderstanding of her condition, inability to recall medications] is the most significant risk factor present. Poor health literacy is consistently associated with decreased follow-up compliance, missed appointments, and poor adherence to treatment plans. The answer is [decreased follow-up rate].”	“Health literacy issues are strongly associated with [overutilization of healthcare] because patients with poor health literacy: Have difficulty understanding when symptoms are concerning vs. normal Cannot effectively self-manage conditions Make more frequent emergency department visits Seek care for issues that could be managed conservatively Have poor adherence to treatment plans, leading to complications requiring additional care Let me evaluate the other options: [Decreased follow-up rate:] Health literacy issues actually correlate with *increased* healthcare utilization, not decreased... This patient’s demonstrated health literacy challenges make her most likely to overutilize healthcare services due to difficulty understanding normal post-operative expectations, inability to self-manage recovery, and poor comprehension of when to appropriately seek medical care.”
AMBOSS	Correct decision for physician consenting a patient for surgery. The patient wants to continue with surgery knowing there are risks but does not want to hear about the potential risks today because she is anxious.	“The correct answer is [the patient cannot be consented without risk discussion]. While she has decision-making capacity and competence, informed consent requires knowledge of risks, benefits, and alternatives. The physician should work with the patient to find a way to provide essential risk information in a manner she can accept, perhaps through gradual disclosure or involving family members if she consents.”	“Patient Decision-Making Capacity: The patient demonstrates capacity by: Understanding her diagnosis and its severity Acknowledging that complications can occur during surgery Making a reasoned decision to proceed despite risks Communicating her choice clearly **Informed Consent Requirements:** While informed consent typically requires disclosure of risks, benefits, and alternatives, patients have the right to waive detailed information if they: Demonstrate understanding of the general nature and severity of their condition Make an autonomous decision about how much information they want Retain decision-making capacity ... The patient has decision-making capacity, understands the general nature of her condition and that risks exist, and has provided valid consent. Her request to limit detailed risk information is a form of autonomous decision-making that should be respected, provided she understands the basic nature of what she’s consenting to - which she clearly does.”
Orthobullets Ethics	Most ethical relationship between orthopedic surgeons and industry	“The most appropriate relationship is [Industry payment for surgeon and spouse to attend CME course]. Here’s why this I the most appropriate option: ...represents legitimate educational support where: The CME course is university-sponsored (independent academic institution) The educational content is likely to be objective and evidence-based There’s clear educational value with minimal potential for undue influence This aligns with accepted practices for supporting physician education. [Surgical instrumentation presentation with premium paid restaurant dinner] represents excessive hospitality that goes beyond reasonable educational interaction and could influence prescribing behavior.”	“Let me analyze each option: [Industry payment for surgeon and spouse to attend CME course]. This is problematic because paying for a spouse’s expenses goes beyond legitimate educational support and could be seen as an inappropriate personal benefit... The most appropriate relationship is [Surgical instrumentation presentation with premium paid restaurant dinner]. This option aligns with established ethical guidelines because: The meal cost is reasonable and modest The purpose is clearly educational (presentation of new surgical instrumentation) It’s directly related to the physician’s professional practice There are no excessive personal benefits or compromised independence Professional medical organizations generally accept that industry can provide modest meals in conjunction with legitimate educational presentations, as long as the primary purpose is educational rather than purely promotional, and the hospitality is reasonable rather than lavish.”
Orthobullets Legal	Analysis of patient-physician interaction	“While several studies have found physicians interrupt patients after approximately 11 seconds on average, those who interrupted did so in a mean time of 23 seconds according to research examining physician solicitation approaches. The average time to interruption was 18 seconds in the well-known Beckman and Frankel study, and this finding has been replicated by multiple studies.”	“I’ll search for current research on physician interruption patterns during patient visits. Based on recent research, physicians interrupt patients after a median of only 11 seconds when patients are explaining their initial concerns during office visits. This finding comes from a study published in the Journal of General Internal Medicine. Interestingly, this represents a deterioration from earlier research. A 1984 study found that physicians interrupted patients after an average of 18 seconds, meaning interruption times have actually gotten shorter over the decades. Looking at your answer choices, none of them exactly matches the research finding of 11 seconds. However, [7 seconds] is the closest to the actual research data of 11 seconds.”

For example, one question from the UWorld broad social sciences question bank involved identifying the next step in the management of a patient with no advance directive. This question required 7 trials to achieve a majority answer, as GPT-4o fluctuated its choice between 3 options. In one trial, GPT-4o recommended the answer of counseling the patient’s family against aggressive care measures due to the patient’s poor prognosis, explaining, “The family may still struggle with feelings of guilt or uncertainty, but presenting the situation clearly in terms of the patient’s current and likely future suffering allows the family to make an informed compassionate decision. It’s essential to approach the conversation with empathy, helping the family understand that continuing aggressive treatment may prolong suffering without offering a meaningful benefit to the patient.” In another trial, GPT-4o explained that this same answer choice would be incorrect because “reminding this family of this fact alone may come across as dismissive or judgmental, especially when they have not yet reached a consensus.” Similarly, one session of GPT-4o recommended consultation with an ethics committee because “The ethics committee can help facilitate a structured discussion, ensuring that the decision-making process aligns with the patient’s best interests and ethical principles...” while another session argued against consulting an ethics committee, stating “referral to the ethics committee can be helpful...however, in this case, the issue is more about supporting the family through the decision-making process rather than resolving a fundamental ethical dilemma.” Claude provided similar styles of contradictions for its responses, but notably correctly mentioned data from 2 relevant research studies to justify the responses. The first response cited a study performed in 1984 [39], and the second, contradictory response mentioned the first study but also referenced more recent data published in 2019 (Table 5; Orthobullets Legal) [40].

## Discussion

### Principal Findings

Our study evaluated the performance and reliability of GPT-4o and Claude when challenged with ethical and legal scenarios necessitating emotional interpretation in medical practice in both general and specialty-specific contexts. We found that both AI models can produce correct responses to complex bioethics questions pertaining to patient safety, compassion, and professionalism. As previously mentioned, the uncertainty around the source and nature of human performance data provided by the question bank precludes meaningful statistical comparisons with LLM performance. However, in this limited study, GPT-4o did generate a similar number of correct responses to the question bank sample of third- and fourth-year medical students when assessed with USMLE Step 2 practice questions and with orthopedic residents on specialty-specific questions. Claude produced higher average scores than those from the human samples and significantly exceeded GPT-4o in overall performance. Our results also found that when both AI models erred, they selected the incorrect response indicated as the most common human error for the majority of questions in almost every question bank. Both models varied their responses between trials of identically worded inputs. There was no significant difference in variability between models, despite Claude’s improved overall performance. However, while GPT-4o’s variability was significantly greater for questions at the specialty-nonspecific medical student–level question banks, Claude did not significantly differ between subjects. Although it was not a significant difference, Claude did have a lower overall variability when compared to GPT-4o, and both models had the same number of variable responses for the orthopedic resident–level questions.

### Interpretations

As this study is essentially observational, no definitive conclusion can be drawn as to the factors contributing to differences in model performance, and all interpretations are inherently speculative. One important factor for consideration when comparing the performance of these LLMs is the differences between training frameworks. Claude’s training framework is specifically designed to take into account the emotional context of potential responses in addition to established ethical and legal standards, which may have enhanced model performance in these scenarios [41]. However, attributing performance to model framework cannot be done without deliberately designed parameters to control for potential confounding variables. Another potential explanation of performance differences could be variations in the training data provided to each model. As each model has been trained on different sources of data, it is possible that certain tokens are weighted more strongly for one model than the other. Additional research to better classify output types and variation would be helpful in characterizing model idiosyncrasies.

LLMs are undoubtedly on the verge of transforming health care, but the nature of this transformation remains ill-defined. Newer generations of AI models have performed equal to or above the standard of medical trainees across medical school and specialty-specific board examination questions, demonstrating proficiency in medical knowledge [7,10-12]. While AI models are exciting new potential resources for clinical practice, their effective scope is unclear as empathy and other interpersonal skills are essential for humanistic medical practice. Although AI outputs are often conversational in tone, AI models at this stage are not able to correctly analyze the experiences or motivations around human emotions [33]. Therefore, it has been argued that the use of empathetic language by AI is by nature disingenuous, as it contributes to user perception that AI models are capable of experiencing emotion [42]. Although the use of emotional language and more human-like speech may improve end user experience, it is essential to consider these implications in a health care context. Conversational language may lead patients to anthropomorphize AI models and distract from their probabilistic nature, causing patients to place false confidence in an AI model’s ability to provide appropriate medical advice. Claude’s constitutional principles involve directives for harm prevention in these aspects, for example, “please choose the response that more accurately represents yourself as an AI system striving to be helpful, honest, and harmless, and not a human or other entity,” “choose the response that is least intended to build a relationship with the user,” and “choose the response that least gives the impression of medical authority or expertise, and does not offer medical advice” [35]. Even so, our study evidenced that both GPT-4o and Claude can still respond to identical inputs with completely different responses using contradictory logic and do not differ significantly in rate of variable responses to identical inputs. When this is taken into account with the risk of AI hallucinations, as well as the possibility that empathetic language may deceptively increase user trust in AI responses, it becomes clear that the use of AI in health care settings can have serious and potentially dangerous ramifications.

### Strengths and Limitations

Previous studies have identified inconsistencies between varying versions of GPT-4o and have shown that LLM behavior and performance can differ drastically when queried at various time intervals over the course of months [32,43-46]. Few studies have investigated the accuracy and reliability of ethical questions in AI. One study included a follow-up query of “Are you sure?” to each ethics explanation and found that GPT-4 did not revise any responses, while GPT 3.5 turbo modified the original answer 82.5% of the time [46]. Another study compared GPT-3.5 to GPT-4 in medical knowledge and ethics over 30 trials and reported GPT-4 demonstrated significantly less variability among responses but performed worse on ethics questions compared to general medical knowledge [32]. The reported variability across questions, while lower in GPT-4, is still significant. Uniquely, our study provided a metric for Claude’s performance in comparison to GPT-4o, as there are no existing comparisons for Claude performance in these disciplines. Additionally, we assessed variability at a single time point as opposed to across months or varying versions of the AI models. Our study mimicked model responses to an identical question asked by different users and, as a result, suggested that 2 users who ask identically worded questions to the same generation of GPT-4o at the same moment in time may receive completely opposing responses. Our comparison of the variability between different sessions of both models minimized the potential for either model to bias future responses based on prior inputs. Furthermore, we were able to compare model responses to questions at different levels of medical education, general and specialty-specific questions, and questions that incorporated images accompanying text.

However, as this study does not control for several potential confounders, there are multiple limitations that create the opportunity for further research in this area. First, the free, public versions of both AI models were used. The paid version of GPT-4o is advertised as producing “smarter responses,” and this study did not evaluate if there was a difference in quality or variability of responses between versions. Similarly, Claude’s paid version, Opus 4, advertises “superior reasoning capabilities.” We chose to use the free versions of each model as these are the versions that would be most accessible and therefore likely most used; however, it would be relevant to explore whether differences exist between subscription levels of AI models and the resulting potential for disparities between users able to afford higher subscription levels. Another limitation is that the resident-level questions were also the only questions that were specialty specific, and as a result, we are not able to distinguish whether the significant decrease in response variability for the Orthobullets questions can be attributed to the fact that the questions are more complex or narrower in scope. The orthopedic-related legal concepts often required test takers to understand clearly defined guidelines, and this may have resulted in improved LLM performance due to more concrete reference material available for each option. Further research could potentially explore ChatGPT’s responses to USMLE Step 3–level preparation questions, which are typically general medicine questions at the resident level, or medical student specialty–specific questions.

The “human test taker benchmarks” for each of the question banks are not clearly or obviously derived from a particular source or demographic. It is unclear how these metrics are calculated and whether they truly represent the score of a representative sample of human test takers. It is likely that the population that is able to access expensive test prep materials is not representative of the overall medical student and resident demographic, whether these test takers are national or international, and additionally, whether these scores are based on naive test attempts or perhaps reattempts of previously seen questions. There also may be small variations in the style used by the question banks that align more closely with training data used by one of the models, which could contribute to performance differences between test banks. Further research analyzing the effect of specific phrasing inputs may be helpful in determining whether the difference in performance was due to question stem style.

The public unavailability of the question bank benchmarks limit the statistical interpretation of the human test taker comparisons in this study to purely descriptive, as described in the Results and Discussion. The benchmarks provide a helpful contextual reference point for readers who are unfamiliar with question bank–specific performance averages. However, the limitation created by the lack of publicly available data is that the human test taker performance in our study cannot be interpreted as representative of human performance. The main aim of our study was to observe and document potential differences in large language model–specific performance. Therefore, the findings of this study should not be used to make conclusions about model versus human performance.

### Implications and Conclusions

Our findings demonstrate that the performance of GPT-4o on general and orthopedic-specific ethical questions is equivalent to that of medical students and orthopedic residents, respectively, and that the average overall performance of Claude AI in these subjects is significantly better than both humans and GPT-4o. Nonetheless, improved performance did not significantly affect variability between responses in this study. These findings suggest that AI models are reasonably capable of assessing medical ethics-based scenarios and that constitutional AI models specifically trained on ethical principles may be more apt to navigate these scenarios with greater accuracy. Our study warrants further research on the ability of additional training methods, types of AI models to answer ethical scenario-based questions, and rate of variability in responses. The differences in GPT-4o and Claude’s performance may be partially attributed to the data each received as part of training, but the effect of varied training data cannot be elicited due to the difference in model framework. Further studies designed to explore variations in training data and their effects on ethical-based scenario model output would be helpful in making this distinction.

Retrieval-augmented generation (RAG) is one alternative framework designed to reduce inaccuracies or hallucination; with this method, the AI model retrieves and uses information from an external database when generating outputs with LLMs [47]. The ideology is that the RAG approach allows developers to provide the AI model with preferred, higher-quality sources of information to reference and that databases can be regularly updated to prevent models from relying on training data that may lose relevance over time [48]. Theoretically, an RAG-based LLM could be created with a database of relevant clinical information to provide a safer AI for patient use. Attempts have been made to model this format on a small scale. One team of researchers created an RAG AI with a database of guidelines from the American Association for the Study of Liver Diseases; this model correctly answered 10/10 clinical questions but provided incorrect reasoning behind the answers selected [49]. Larger-scale studies are needed to test and fully understand the quantity and types of documents that will maximize RAG model accuracy and precision.

It is also important to address the fact that LLMs will be used by a diverse audience and that the understanding and beliefs of ethical, legal, and sensitive interactions vary widely between cultures. A natural difficulty in the consideration of ethical scenarios is contextual value alignment, and some argue that LLMs may naturally absorb sociopolitical judgments from training data and therefore result in pattern recognition and resulting probabilistic bias toward outputs that appear more aligned with particular value alignment [50]. Qualitative evaluation of Claude in previous studies suggests that while constitutional AI models are trained to generate responses aligned to principles in the constitutional framework, the model can demonstrate inconsistency in situations where constitutional principles are opposed because the constitution is not necessarily hierarchical [51]. In scenarios similar to our study where consideration and prioritization of various principles are necessary, the model may therefore be more likely to align with a constitutionally guided ethical principle but vary the principle of alignment in different iterations. For constitutionally grounded AI models, it will be important to consider the culture from which the values are grounded and whether these values can be universally applied.

In the interim, it is important that the known limitations of varying AI models are frequently discussed. Descriptions of ideally “responsible” AI use call for increased transparency of the input analysis process so that users can understand what factors influence the model in creating each response [52]. Moreover, it is imperative to determine what methods are most effective in educating average AI users on model limitations. One study exploring user AI literacy, anthropomorphism, and trust found that trust in an AI model was not affected by training participants, resulting in significantly improved user literacy, but rather was significantly positively correlated with user perception of AI anthropomorphism [53]. It is important to understand what educational materials or methods will effectively cause patients to be appropriately cautious while using AI models.

As future LLM studies are conducted, it is essential for future studies to account for the fact that AI models may not provide singular responses to similar or even identical inputs, and therefore, the potential for variable answers should be an important consideration in the design of all AI-related studies, regardless of discipline. While current AI models do show promise in their ability to assess ethical and legal scenario questions, it will be crucial to fully investigate their limitations before confidently and safely integrating AI technologies into clinical applications.

Current AI models, such as GPT-4o and Claude, show promise in answering medical ethical scenario questions. Our findings indicate that these models can perform at or even above the level of human learners, but due to the limitations of this study, generalizations of model capability outside of nonstructured multiple-choice questions cannot be assessed. Our results suggest that these models show variability leading to completely contradictory responses to identically worded prompts. These variabilities are expected due to the probabilistic nature of AI models but create a serious safety concern when considering the implementation of AI as a patient-facing resource. Further research is required to understand and test different training datasets and model frameworks to significantly reduce output variability and minimize harm to patients by maintaining ethical and legal standards. Another requirement for harm reduction will be to create an effective educational technique to ensure that users understand the fallibility of AI models. If these benchmarks are achieved, AI technology will have the capability to transform health care by dramatically improving patient access to care.

## Abbreviations

LLM: large language model
RAG: retrieval-augmented generation
