# Correction: Performance of GPT-4o and Claude in Medical Ethics Scenarios: Comparative Study

**DOI:** 10.2196/113933

**Published:** 2026-11-11

**Authors:** Karishma R Desai, Anna L Gorsky, Nicole A Zelenski

**Affiliations:** 1 Division of Hand Surgery, Department of Orthopaedic Surgery, Emory University, 80 Jesse Hill Jr Drive SE, Atlanta, GA, 30303, United States, 1 7045268123

In “Performance of GPT-4o and Claude in Medical Ethics Scenarios: Comparative Study” [1], the authors noted 3 errors. The below changes have been made.

First, Table 2 incorrectly listed the denominator for the GTP-4o UWorld Interpersonal-Specific questions as 11; the correct denominator is 10. The AMBOSS numerator and denominator for the GPT-4o questions were listed as 1/2 and have been corrected to 9/18. The revised table is presented as Table 1 below, with changes formatted in italics.

**Table 1.** Incorrectly answered questions per question set for which GPT-4o and Claude selected the incorrect answer most commonly chosen by human test takers.

Question set	GPT-4o^a^, n/N (%)	Claude^b^, n/N (%)
UWorld All Social Sciences	6/13 (46)	5/7 (71)
UWorld Interpersonal-Specific	*9/10 (90)*	4/4 (100)
AMBOSS	*9/18 (50)*	3/4 (75)
Orthobullets Ethics	6/7 (86)	4/4 (100)
Orthobullets Legal	7/12 (58)	1/1 (100)

a Total: 37/60 (62%).

b Total: 17/20 (85%).

Second, the comparison between incorrect responses was mistakenly listed as significant during the extensive recalculation of statistics; the *χ*^2^ value of this test (*P*=.03) was incorrectly used instead of the previously used and more appropriate Fisher exact interpretation (*P*=.07). Therefore, a few manuscript sections were amended.

In the Results section of the Abstract, the following sentence was revised:

*GPT-4o selected the incorrect response for 60 (30%) total questions and chose the incorrect response most commonly selected by humans significantly more frequently on UWorld interpersonal-specific questions (30/40, 75%) than on UWorld all social sciences (27/40, 68%; P=.03).*

The sentence now reads:

*Neither GPT-4o or Claude showed a significant difference in the rate of most common incorrect response selection between question categories.*

The following sentence from the Results was revised:

*Analysis did show a significant difference in the frequency that GPT-4o chose the most popular incorrect answer between both UWorld general social sciences and interpersonal-specific sets (P=.03), but no difference between the UWorld (general and interpersonal) and AMBOSS (legal medicine and ethics) sets (P=0.33), Orthobullets Ethics and Legal sets (P=.22), or medical student general and orthopedic resident–level (UWorld/AMBOSS and Orthobullets) sets (P=.46; Table 2).*

The sentence now reads:

*Analysis did not show a significant difference in the frequency that GPT-4o chose the most popular incorrect answer between both UWorld general social sciences and interpersonal-specific sets (P=.07), UWorld (general and interpersonal) and AMBOSS (legal medicine and ethics) sets (P=.33), Orthobullets Ethics and Legal sets (P=.22), or medical student general and orthopedic resident–level (UWorld/AMBOSS and Orthobullets) sets (P=.46; Table 2).*

In the Implications and Conclusions section of the Discussion, the following sentences were revised:

*Further studies designed to explore variations in training data and their effects on ethical-based scenario model output would be helpful in making this distinction. For instance, it is not clear why GPT-4o chose the most popular incorrect answer significantly more frequently on the UWorld interpersonal questions than on UWorld Social Sciences, but Claude, which is designed to be more conversational, did not.*

The revised sentences now read:

*Further studies designed to explore variations in training data and their effects on ethical-based scenario model output would be helpful in making this distinction.*

Lastly, the editor requested that a statement be provided as to whether any limitations to the scope of the article are created due to the public unavailability of the question bank benchmarks. The following paragraph was added to the end of the Strengths and Limitations section of the Discussion:

*The public unavailability of the question bank benchmarks limit the statistical interpretation of the human test taker comparisons in this study to purely descriptive, as described in the Results and Discussion. The benchmarks provide a helpful contextual reference point for readers who are unfamiliar with question bank–specific performance averages. However, the limitation created by the lack of publicly available data is that the human test taker performance in our study cannot be interpreted as representative of human performance. The main aim of our study was to observe and document potential differences in large language model–specific performance. Therefore, the findings of this study should not be used to make conclusions about model versus human performance.*

Fortunately, these corrections have only a minor effect on the results as reported and do not alter the overall interpretation or conclusions of the manuscript. We appreciate the efforts of the editorial office in identifying these errors, allowing us the opportunity for correction, and confirming that the findings of the article are not affected.

The correction will appear in the online version of the paper on the JMIR Publications website together with the publication of this correction notice. Because this was made after submission to PubMed, PubMed Central, and other full-text repositories, the corrected article has also been resubmitted to those repositories.
